# Cognitive-motor dual task induces strategic kinematic adjustments with increased toe-obstacle clearance for older adults during obstacle crossing

**DOI:** 10.1371/journal.pone.0334321

**Published:** 2025-10-14

**Authors:** Shiuan-Huei Lu, Yi-Chun Kuan, Kuan-Wen Wu, Ting-Ming Wang, Tung-Wu Lu

**Affiliations:** 1 Department of Biomedical Engineering, National Taiwan University, Taipei, Taiwan, ROC; 2 Taipei Neuroscience Institute, Taipei Medical University, Taipei, Taiwan, ROC; 3 Department of Neurology, School of Medicine, College of Medicine, Taipei Medical University, Taipei, Taiwan, ROC; 4 Dementia Center and Department of Neurology, Taipei Medical University Shuang Ho Hospital, New Taipei City, Taiwan, ROC; 5 Department of Orthopaedic Surgery, School of Medicine, National Taiwan University, Taipei, Taiwan, ROC; 6 Department of Orthopaedic Surgery, National Taiwan University Hospital, Taipei, Taiwan, ROC; Drexel University School of Biomedical Engineering Science and Health Systems, UNITED STATES OF AMERICA

## Abstract

**Background and objectives:**

Falls among older adults, especially during obstacle crossing, lead to severe outcomes like fractures and higher healthcare costs due to declining cognitive and motor functions. The study aimed to quantify the kinematic adjustments at individual joints and end-points of the pelvis-leg apparatus in older adults during cognitive-motor dual task involved crossing obstacles of varying heights while performing serial subtraction.

**Methods:**

Sixteen healthy older adults each walked and crossed obstacle of three varying heights with leading and trailing limb under single-task and dual-task conditions. Toe-obstacle clearances and pelvic and lower limb angular motions were calculated. Two-way analyses of variance were conducted to study within-subject (task and height) effects on the variables.

**Results:**

Older adults showed significantly reduced crossing speed and increased leading and trailing toe-obstacle clearances. During dual-task obstacle crossing, there were increased pelvic anterior tilt, swing hip abduction and knee flexion, but decreased stance hip adduction at leading-limb crossing, compared to single-task (p < 0.05). There were increased in pelvic posterior tilt and swing knee flexion, but decreased pelvic upward list, stance hip adduction and stance knee flexion during dual-task obstacle crossing at trailing-limb crossing (p < 0.05).

**Discussion and implications:**

The study found healthy older adults showed reduced crossing speed and adapting behaviour, with distinct kinematic changes at the pelvis, hip, and knee joints, leading to increased toe-obstacle clearances. While this may affect balance adversely. To mitigate fall risks, older adults should consider balance training and avoid distractions like phone use during obstacle crossing. Future studies should explore unexpected obstacles and its effects on at-risk populations.

## Introduction

Falls among older adults is a significant public health issue, with potentially severe consequences such as fractures, loss of independence, and increased healthcare costs [1,2]. Among the various contexts in which falls occur, obstacle-crossing is particularly challenging due to its demands on motor planning and attentional resources [3]. Obstacle-crossing depends heavily on the central nervous system (CNS)’s higher-level processes [4] to coordinate motor and cognitive functions, including integrating visual information, spatial awareness and decision-making for successful obstacle navigation [5]. Natural ageing or impairment in cognitive functions or available attentional resources can significantly increase the risk of falls during obstacle-crossing. In particular, ageing is associated with decreased attentional control and cognitive flexibility, heightening vulnerability to the adverse effects of divided attention, and the ability to simultaneously focus on multiple tasks, during complex motor tasks such as obstacle-crossing [6,7]. Divided attention is crucial for assessing obstacle parameters, adjusting step length and trajectory, and monitoring environmental cues for potential hazards [8], with consequences on the lower-limb kinematics during movement control and execution [9]. Age-related decline in neuromuscular efficiency of the lower limbs and responsiveness may lead to slower reaction time, diminished balance and thus, a greater likelihood of falls during obstacle crossing [10,11]. Therefore, identifying the effects of divided attention on the lower limb kinematic strategies older adults adopt during crossing obstacles can aid in creating strategies to lower fall risks in this demographic.

Tripping, imbalance and failure to regain equilibrium while crossing obstacles are prominent factors causing falls in older adults [12,13]. To cross obstacles safely, older adults control the motion of their lower-limb joints, ensuring sufficient foot-obstacle clearance while maintaining dynamic balance. Older adults were found to cross obstacles with enhanced clearance of the leading toe-obstacle and trailing toe-obstacle distance but reduced leading heel-obstacle distance in contrast to young adults [11]. Such a crossing strategy prioritizes foot-obstacle clearance to lower the tripping risk, albeit at the cost of increased energy expenditure [11]. Another common factor that affects the fall risk during obstacle crossing in the older population is when the attention is divided into performing dual tasks, common in our daily activities, such as crossing obstacles while talking to friends or mobile phone or being distracted by other events [7,14]. With the decline of divided attention and the increase in possible occurrence of cognitive-motor interference with age, dual tasks pose an increased risk of falls in the elderly [15]. Any associated alterations of the joint kinematics will impact the kinematic coordination between joints and their end-point, essential for successful obstacle-crossing. On the other hand, older adults may develop adaptative strategies to help compensate for age-related declines in attentional control and cognitive flexibility, coordinate movement, and adjust to environmental obstacles.

Cognitive-motor interference occurs when a cognitive task and a motor task are performed simultaneously, often referred to as a dual task, which can impair the performance of one or both tasks [16]. This interference is particularly pronounced when the cognitive task is demanding, as it can significantly disrupt overall motor performance [17,18]. Given the brain’s limited pool of processing resources, dual-tasking reduces the amount of resources available for each task, potentially making performance deviations or adjustments more detectable compared to single-task conditions [19]. Previous studies using cognitive-motor dual-task paradigms have found that older people require more attentional resources during gait than young adults [20]. Simultaneously performing a cognitive-motor dual task can compromise the available attentional resources and thus increase the risk of falling [21]. During obstacle crossing, older adults showed reduced gait speed when concurrently performing a cognitive task [22]. Older adults adopt slower gait speeds as an adaptive strategy to improve stability when their cognitive resources are stretched [23]. However, previous studies on older adults during dual-task obstacle crossing measured only temporospatial parameters, so how the reduced crossing speed affected the three-dimensional motions of the lower-limb kinematic strategies remained unclear [23,24].

Studies on the inter-joint and joint-to-end-point kinematic relationships in the pelvis-leg apparatus have revealed the kinematic changes to cross obstacles across different populations [25–28]. Taking the human pelvis-leg apparatus as a multi-link system, changing the angle of one joint may prompt adjustments in adjacent joints, influencing the end-point position of the swing limb. For example, increased hip flexion has been observed in older adults to increased toe-obstacle clearance [29], reducing tripping risks [11]. By synthesizing the kinematic changes using the multi-link system approach, kinematic relationships between individual joints and end-points can be mapped across various groups of subjects and types of motor tasks, indicating the neuromusculoskeletal or attentional control within each groups. However, no study has documented the postural and end-point kinematic changes during dual-task obstacle crossing in older adults.

The objective of the current study was to quantify the kinematic adjustments of individual joints and end-points of the pelvis-leg apparatus for older adults during cognitive-motor dual task involved crossing obstacles of varying heights while performing serial subtraction. By analyzing the joint- and end-point kinematic changes across different obstacle heights under both for single- and dual-task conditions, the study identified the kinematic strategies adopted by the older adults. It was hypothesised that older adults would have increased toe-obstacle clearance through strategic kinematic adjustments during obstacle-crossing under cognitive-motor dual-task conditions.

## Materials and methods

### Ethics statement

This study was approved by the Taipei Medical University Joint Institutional Review Board (Permit No. N201903100) and was conducted following the Declaration of Helsinki of the World Medical Association. All participants provided written informed consent and were fully informed of the study procedures prior to the study.

### Participants

Sixteen healthy older adults (age: 68.4 ± 4.4 years; body height: 156.2 ± 6.8 cm; body mass: 56.7 ± 10.6 kg) were recruited between September 1, 2019 and December 31, 2021. All participants underwent a comprehensive neuropsychological assessment, including the Cognitive Abilities Screening Instrument, Digit Span subtest from the Wechsler Adult Intelligence Scale, Word Sequence Learning Test and verbal fluency, to confirm the absence of impaired cognitive functions [30–32]. A participant would be excluded if they were functionally dependent, could not walk independently, had difficulties in effective communication, had severe uncorrected visual or auditory impairments, had a CNS lesion or severe neuromusculoskeletal disorders, or underwent lower limb surgeries that could affect their gait.

### Experimental protocol

In a gait laboratory, each participant performed obstacle crossing under single-task and dual-task conditions. During single-task obstacle crossing, participants walked along a 10-meter walkway at their preferred walking speed and crossed a lightweight, tube-like obstacle placed horizontally on a metal frame. The obstacle height was adjusted to 10%, 20%, and 30% of each participant’s leg length [11,33]. During dual-task obstacle crossing, participants walked and crossed the same obstacle while concurrently counting down by threes from a randomly generated number between 90 and 100 (e.g., 97, 94, 91, 88…) and spoke aloud. Two infrared-retroreflective markers were placed at each end of the tube to define the position and height of the obstacle. A total of thirty-nine retroreflective markers were attached to typical anatomical landmarks to track the body’s segmental movements of the body in space at 200 Hz, using an 8-camera motion analysis system (Vicon MX T-40 OMG, UK) [34–39], while the ground reaction forces (GRF) were recorded at 1200 Hz using three forceplates (OR6–7, AMTI, USA) positioned on both sides of the obstacle. The starting position of each participant was adjusted by the examiner to ensure that the participant stepped naturally onto the forceplates without visual targeting. Data were collected from three crossing cycles per leading limb, yielding a total of six complete crossing cycles per participant. The sequence of the six condition blocks (2 tasks x 3 obstacle heights) was randomized.

### Data analysis

The crossing speed was calculated by dividing the distance travelled from the mid-point of the two anterior superior iliac spines by the time taken from the leading toe-off to the trailing heel-strike. Toe-obstacle clearance for both limbs was measured vertically from the swing limb’s toe marker to the obstacle when directly above the obstacle. The horizontal distance between the trailing limb’s toe marker and the obstacle during stance before crossing defined the trailing toe-obstacle distance. Similarly, the horizontal distance between the leading limb’s heel marker and the obstacle after crossing was called the leading heel-obstacle distance [40]. To describe the pelvis-leg kinematics during obstacle-crossing, the leading limb was defined as the reference limb, and local coordinate systems were attached to the body segments. The x-axes were directed anteriorly, the y-axes superiorly and the z-axes oriented to the right [27]. The joint angles were calculated as the rotations of the distal segment relative to the proximal following a rotation sequence of z-x-y [41], while those of the pelvis were defined with the reference to the laboratory coordinate system. Pelvic hiking/ pelvic drop indicates that the hip is positioned higher/ lower than the contralateral [28]. In the transverse plane, ipsilateral pelvic rotation indicates that the hip is anterior to the contralateral. To reduce the effects of skin movement artefacts, a global optimization technique was used to minimize the weighted sum of squared distances between observed and calculated marker positions subject to joint constraints [42]. For the statistical analysis, values of the crossing angles, i.e., joint angles when the crossing toes were above the obstacle, were obtained [43].

### Statistical analysis

A two-way repeated measures analysis of variance (ANOVA) was conducted to compare between task conditions (single-task versus dual-task) and between obstacle heights (10, 20, and 30% of leg length) for the crossing speed, each end-point parameter, and all calculated pelvis-leg kinematic variables. For each variable, data were averaged across crossing cycles, and then averaged across both sides for each subject. A Shapiro-Wilk test confirmed that all calculated variables exhibited a normal distribution, and the homogeneity of variance across groups was verified using Levene’s test. A significance level of 0.05 was set for all test conditions. All statistical analyses were conducted using SPSS version 20 (SPSS Inc., Chicago, IL, USA). The multi-link system approach was applied to synthesize significant joint and end-point kinematic adjustments [25–28,44,45], identifying the kinematic crossing strategies adopted by older adults under single-task and dual-task conditions.

An *a priori* power analysis based on pilot results using GPOWER [46], indicated that, for two-way repeated measures analysis of variance (ANOVA), a projected sample size of twelve subjects per group would achieve a power of 0.8, detecting a large effect size (Cohen’s f = 0.4) at a significance level of 0.05. Therefore, 16 subjects were considered adequate for the current study.

## Results

The dual-task obstacle-crossing condition provoked an overall decreased crossing speed but increased leading and trailing toe-obstacle clearances compared to those during a single task (Table 1).

**Table 1:** Means (standard deviations) of the crossing speeds and end-point variables of healthy older adults when crossing obstacles of heights of 10%, 20% and 30% of the subjects’ leg length (LL) during single task and dual task. P-values for main task and height effects are given as no interactions were found.

Variables	Obstacle height (%LL)	Older adults		Main Effect
		Single Task	Dual Task	P_T,_ P_H_
Crossing speed (m/s)	10	0.90 (0.12)	0.81 (0.14)	0.003*, < 0.001↓
	20	0.81 (0.12)	0.77 (0.12)	
	30	0.76 (0.12)	0.71 (0.10)	
Leading toe- obstacle clearance (%LL)	10	19.7 (5.0)	21.2 (5.3)	<0.001*, 0.003↑
	20	20.4 (4.4)	23.8 (4.9)	
	30	21.9 (4.0)	24.4 (4.6)	
Trailing toe- obstacle clearance (%LL)	10	19.1 (8.9)	21.6 (9.6)	0.001*, 0.003↑
	20	21.0 (10.1)	24.9 (9.6)	
	30	23.2 (7.0)	26.2 (7.6)	
Leading heel-obstacle distance (mm)	10	150.8 (43.4)	151.8 (35.1)	0.219, 0.031↓
	20	145.1 (40.9)	135.8 (37.8)	
	30	135.6 (40.6)	129.3 (35.2)	
Trailing toe-obstacle distance (mm)	10	187.1 (29.6)	183.1 (24.1)	0.406, 0.110
	20	187.8 (30.2)	196.5 (27.9)	
	30	194.7 (26.9)	199.1 (34.6)	

P_T_ = Single task vs. Dual task; P_H_ = *p*-value for obstacle height; * indicates a significant task effect (P_T_ < 0.05); ↑ indicates a linearly increasing trend and ↓ indicates a linearly decreasing trend (P_H _< 0.05).

**Fig 1:**
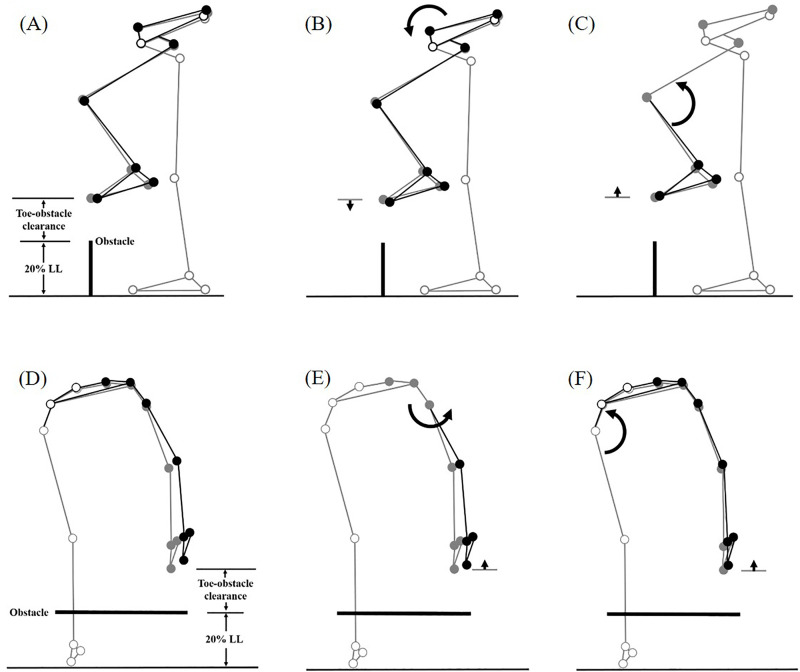
Effects of the observed significant angular changes at individual joints on the leading toe-obstacle clearance in healthy older adults during dual-task obstacle crossing (black stick figure) compared with the mean single-task obstacle crossing (grey stick figure) at the obstacle heights of 20% leg length. The stick model was generated using marker positions from a typical older adult participant. Segments with solid grey circles are joints of the reference limb. With the stance foot fixed to the ground, each joint was independently rotated based on the mean angular change reported in Tables 2 and 3, while keeping the angles of the other joints fixed. The segments of the stance limb and the segments of the swing limb distal to the current joint stationary. Dual task provoked the increased pelvic anterior tilt (B), and increased knee flexion (C), increased swing hip abduction (E), but decreased stance hip abduction (F). As illustrated by the black stick figure, while (C), (E) and (F) contributed to an increase in leading toe-obstacle clearance, (B) had the opposite effect. The combined result of these changes at the same time led to the overall increase in leading toe-obstacle clearance as compared to single task.

**Fig 2:**
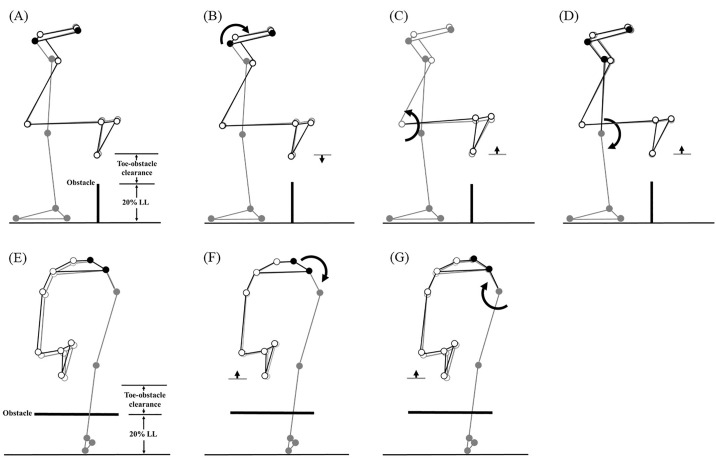
Effects of the observed significant angular changes at individual joints on the trailing toe-obstacle clearance in healthy older adults during dual-task obstacle crossing (black stick figure) compared with the mean single-task obstacle crossing (grey stick figure) in the obstacle heights of 20% leg length. The stick model was generated using marker positions from a typical older adult participant. Segments with solid grey circles are joints of the reference limb. With the stance foot fixed to the ground, each joint was independently rotated based on the mean angular change reported in Tables 2 and 4, while keeping the angles of the other joints fixed. The segments of the stance limb and the segments of the swing limb distal to the current joint stationary. Dual task provoked the increased pelvic posterior tilt (B) and knee flexion in the swing limb (C), but decreased stance knee flexion (D), pelvic upward list (F) and stance hip adduction (G). As illustrated by the black stick figure, (C), (D), (F) and (G) contributed to an increase in trailing toe-obstacle clearance, while (B) had the opposite effect. The combined result of these changes when the trailing toe above the obstacle led to an overall increase in trailing toe-obstacle clearance compared to the single-task condition.

**Fig 3:**
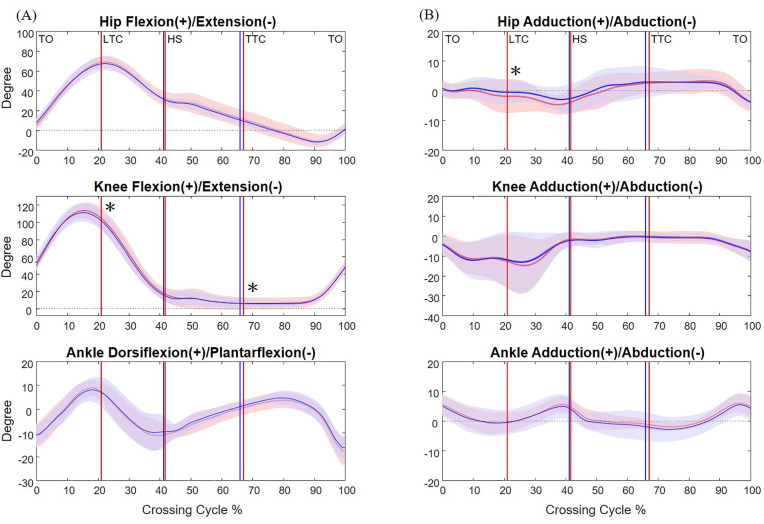
The mean curves of the angles of the hip, knee and ankle joints of the leading limb in the sagittal (A) and frontal plane (B) in healthy older adults during single task (blue) and dual task (red) when crossing obstacles of 20% of leg length. (TO: toe-off of the leading limb; LTC: leading toe above the obstacle; HS: heel-strike of the leading limb; TTC: trailing toe above the obstacle; *: significant task effects, *p* < 0.05).

**Fig 4:**
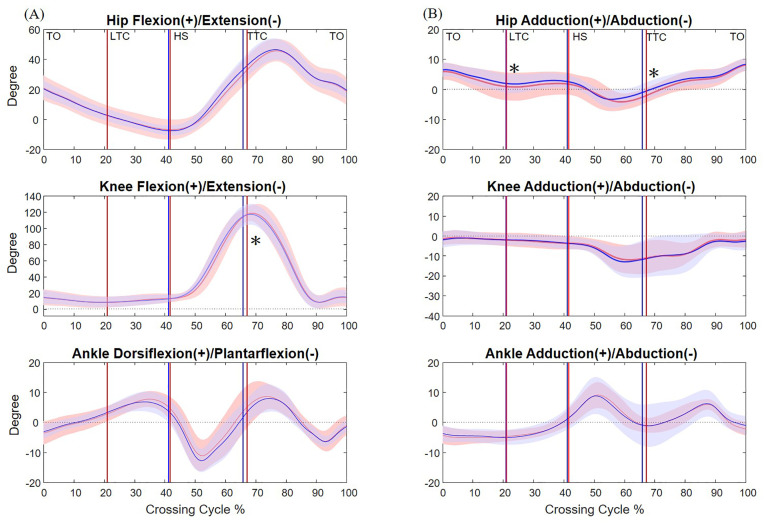
The mean curves of the angles of the hip, knee and ankle joints of the trailing limb in the sagittal (A) and frontal plane (B) in healthy older adults during single task (blue) and dual task (red) when crossing obstacles of 20% of leg length. (TO: toe-off of the leading limb; LTC: leading toe above the obstacle; HS: heel-strike of the leading limb; TTC: trailing toe above the obstacle; *: significant task effects, *p* < 0.05).

**Fig 5:**
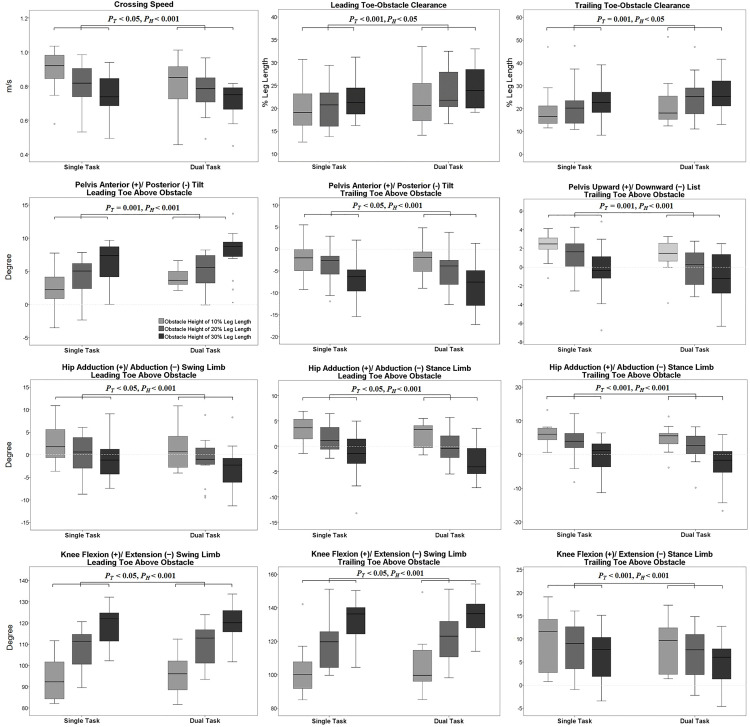
Effects of cognitive-motor dual task (single task vs. dual task) and obstacle height (10%, 20% and 30% of the subjects’ leg length) on the crossing speed, toe-obstacle clearances, pelvic rotations and lower-limb joint angles in the sagittal and frontal planes in healthy older adults during obstacle crossing. Boxplots show the distribution of results, and task and height effects (*P*_*T*_: *p*-values for task factor; *P*_*H*_: *p*-values for height factor) on each variable. Obstacle heights are represented by three grayscale tones (light: 10%, medium: 20%, dark: 30% of leg length).

Significant angular adjustments in individual joints showed different effects on the clearances of both the leading and trailing toe-obstacle for healthy older adults during dual-task obstacle crossing compared to a single task. Some adjustments increased toe-obstacle clearance, while others had the opposite effects (Figs 1 and 2). Compared to single task, healthy older adults showed significantly increased pelvic anterior tilt, hip abduction and knee flexion in the swing limb, alongside decreased hip adduction in the stance limb when the leading toe crossed above the obstacle during dual-task obstacle crossing (Figs 1, 3–5 and Tables 2 and 3).

**Table 2:** Means (standard deviations) of the crossing angles of the pelvis relative to the global norm in healthy older adults when the leading or trailing toe was above the obstacles of heights of 10%, 20% and 30% of the subjects’ leg length (LL) during single task (ST) and dual task (DT). P-values for main task and height effects are given as no interactions were found.

Variables	Task	Obstacle height (%LL)			Main Effect
		10	20	30	P_T_, P_H_
**Leading toe above obstacle**					
Upward (+)/downward (−) list	ST	4.1 (2.1)	5.4 (5.3)	10.5 (6.3)	0.265, < 0.001↑
	DT	5.2 (2.5)	8.3 (3.1)	12.0 (5.9)	
Ipsilateral (+)/ contralateral (-) rotation	ST	−3.0 (4.1)	−3.9 (5.5)	−6.3 (5.1)	0.054, < 0.020↑
	DT	−4.3 (5.5)	−6.5 (6.5)	−8.4 (9.1)	
Anterior (+)/posterior (−) tilt	ST	2.4 (3.1)	4.3 (2.6)	6.3 (3.0)	0.001*, < 0.001↑
	DT	3.4 (2.6)	5.2 (2.5)	7.8 (3.3)	
**Trailing toe above obstacle**					
Upward (+)/downward (−) list	ST	2.3 (1.3)	1.2 (1.9)	−0.4 (2.8)	<0.001^*^, < 0.001↓
	DT	1.3 (1.7)	−0.1 (1.9)	−1.2 (2.7)	
Ipsilateral(+)/ contralateral (-) rotation	ST	−5.3 (3.7)	−5.5 (8.0)	−12.6 (5.7)	0.345, < 0.001↑
	DT	−6.1 (4.3)	−8.8 (6.0)	−16.6 (8.2)	
Anterior (+)/posterior (−) tilt	ST	−2.5 (3.7)	−3.8 (3.9)	−7.1 (4.5)	0.034*, < 0.001↑
	DT	−2.6 (3.5)	−4.9 (4.3)	−8.2 (5.4)	

P_T_ = Single task (ST) vs. Dual task (DT); P_H_ = *p*-value for obstacle height; * indicates a significant task effect (P_T_ < 0.05); ↑ indicates a linearly increasing trend and ↓ indicates a linearly decreasing trend (P_H _< 0.05); Upward list indicates that the contralateral hip joint center is higher than the ipsilateral hip; Ipsilateral rotation indicates that the ipsilateral hip joint center is anterior to the contralateral hip.

**Table 3:** Means (standard deviations) of the crossing angles of the pelvis and those at the hip, knee and ankle joints of the leading swing limb and trailing stance limb in healthy older adults when the leading toe was above the obstacle of heights of 10%, 20% and 30% of subjects’ leg length (LL) during single task (ST) and dual task (DT). P-values for main task and height effects are given as no interactions were found.

Variables	Task	Obstacle height (%LL)			Main Effect
		10	20	30	P_T_, P_H_
**Leading swing limb**					
Hip					
Flexion (+)/ extension (−)	ST	58.7 (6.4)	67.1 (6.0)	74.6 (5.5)	0.220, < 0.001↑
	DT	59.1 (6.8)	68.9 (6.6)	74.7 (5.3)	
Adduction (+)/ abduction (−)	ST	2.7 (4.0)	−0.4 (4.3)	−1.1 (4.6)	0.019*, < 0.001↑
	DT	1.3 (4.3)	−1.1 (4.7)	−2.9 (4.8)	
Knee					
Flexion (+)/ extension (−)	ST	93.4 (9.9)	108.3 (9.2)	118.9 (9.1)	0.031*, < 0.001↑
	DT	95.5 (9.2)	110.1 (9.3)	119.6 (8.5)	
Adduction (+)/ abduction (−)	ST	−12.2 (11.2)	−11.1 (13.9)	−10.1 (16.3)	0.458, 0.333
	DT	−12.6 (11.9)	−11.8 (15.2)	−10.4 (16.0)	
Ankle					
Dorsiflexion (+)/ plantarflexion (−)	ST	9.1 (4.6)	9.4 (4.0)	10.7 (5.3)	0.498, 0.007↑
	DT	8.0 (3.4)	9.5 (4.2)	10.8 (4.5)	
Adduction (+)/ abduction (−)	ST	0.5 (3.7)	0.4 (3.8)	0.1 (4.4)	0.241, 0.544
	DT	0.9 (3.8)	0.4 (3.8)	0.7 (4.6)	
**Trailing stance limb**					
Hip					
Flexion (+)/ extension (−)	ST	2.6 (4.1)	2.9 (4.2)	3.1 (4.4)	0.972, 0.101
	DT	2.1 (3.6)	3.1 (4.3)	3.4 (4.0)	
Adduction (+)/ abduction (−)	ST	3.4 (2.6)	1.5 (2.7)	−1.8 (4.5)	0.002*, < 0.001↓
	DT	2.4 (2.5)	−0.04 (3.1)	−3.3 (3.4)	
Knee					
Flexion (+)/ extension (−)	ST	10.0 (6.4)	9.9 (6.0)	9.6 (6.4)	0.113, 0.664
	DT	9.0 (6.1)	9.7 (6.1)	9.7 (6.3)	
Adduction (+)/ abduction (−)	ST	−1.9 (2.7)	−2.0 (2.7)	−2.3 (2.9)	0.930, 0.002↑
	DT	−1.5 (2.6)	−2.1 (2.8)	−2.6 (3.1)	
Ankle					
Dorsiflexion (+)/ plantarflexion (−)	ST	9.2 (4.6)	9.4 (4.0)	10.7 (5.3)	0.498, 0.007↓
	DT	8.1 (3.4)	9.5 (4.2)	10.8 (4.5)	
Adduction (+)/ abduction (−)	ST	0.5 (3.7)	0.4 (3.8)	0.1 (4.4)	0.241, 0.544
	DT	0.9 (3.8)	0.4 (3.8)	0.7 (4.6)	

P_T_ = Single task (ST) vs. Dual task (DT); P_H_ = *p*-value for obstacle height; * indicates a significant task effect (P_T_ < 0.05); ↑ indicates a linearly increasing trend and ↓ indicates a linearly decreasing trend (P_H _< 0.05).

**Table 4:** Means (standard deviations) of the crossing angles of the pelvis and those at the hip, knee and ankle joints of the trailing swing limb and leading stance limb in healthy older adults when the trailing toe was above the obstacle of heights of 10%, 20% and 30% of subjects’ leg length (LL) during single task (ST) and dual task (DT). P-values for main task and height effects are given as no interactions were found.

Variables	Task	Obstacle height (%LL)			Main Effect
		10	20	30	P_T_, P_H_
**Trailing swing limb**					
Hip					
Flexion (+)/ extension (−)	ST	26.4 (6.0)	30.7 (6.4)	34.5 (6.9)	0.113, < 0.001↑
	DT	26.7 (5.9)	31.3 (6.3)	37.6 (10.7)	
Adduction (+)/ abduction (−)	ST	−0.02 (3.6)	−0.04 (3.4)	−0.4 (3.5)	0.057, 0.481
	DT	−0.7 (3.7)	−1.2 (4.1)	−0.9 (4.6)	
Knee					
Flexion (+)/ extension (−)	ST	102.2 (14.5)	118.0 (14.4)	131.7 (11.8)	0.003*, < 0.001↑
	DT	105.2 (15.8)	122.2 (14.2)	134.7 (10.9)	
Adduction (+)/ abduction (−)	ST	−12.1 (7.7)	−11.9 (8.4)	−11.2 (7.7)	0.422, 0.229
	DT	−12.2 (8.7)	−10.9 (8.1)	−11.1 (7.5)	
Ankle					
Dorsiflexion (+)/ plantarflexion (−)	ST	−1.5 (7.3)	0.9 (7.1)	6.5 (7.7)	0.381, < 0.001↑
	DT	−1.3 (5.9)	5.8 (5.8)	9.4 (6.5)	
Adduction (+)/ abduction (−)	ST	0.1 (5.7)	−0.2 (6.3)	1.1 (6.7)	0.472, 0.431
	DT	0.6 (5.2)	0.1 (5.6)	1.3 (6.5)	
**Leading stance limb**					
Hip					
Flexion (+)/ extension (−)	ST	11.5 (4.4)	10.7 (3.6)	11.4 (5.1)	0.862, 0.072
	DT	10.7 (4.5)	10.8 (4.4)	10.8 (4.0)	
Adduction (+)/ abduction (−)	ST	6.0 (2.8)	3.4 (4.9)	0.2 (4.8)	<0.001*, < 0.001↓
	DT	4.8 (3.5)	2.3 (4.5)	−2.8 (6.1)	
Knee					
Flexion (+)/ extension (−)	ST	10.0 (6.0)	8.3 (5.6)	6.0 (5.4)	0.001*, < 0.001↓
	DT	8.3 (5.3)	6.8 (5.2)	5.1 (4.8)	
Adduction (+)/ abduction (−)	ST	−0.6 (2.4)	−0.6 (2.4)	0.4 (2.2)	0.291, 0.991
	DT	−0.2 (2.1)	−0.5 (2.5)	−0.4 (2.1)	
Ankle					
Dorsiflexion (+)/ plantarflexion (−)	ST	1.8 (2.5)	1.6 (2.5)	1.2 (3.1)	0.057, 0.453
	DT	1.3 (2.7)	1.2 (2.9)	0.9 (2.8)	
Adduction (+)/ abduction (−)	ST	−2.3 (2.0)	−1.3 (3.8)	−1.2 (3.1)	0.055, 0.293
	DT	−1.2 (2.4)	−0.6 (2.9)	1.1 (4.3)	

P_T_ = Single task (ST) vs. Dual task (DT); P_H_ = *p*-value for obstacle height; * indicates a significant task effect (P_T_ < 0.05); ↑ indicates a linearly increasing trend and ↓ indicates a linearly decreasing trend (P_H _< 0.05).

When the trailing toe crossed above the obstacle, significantly increased pelvic posterior tilt and knee flexion in the swing limb, but decreased pelvic upward list, hip adduction and knee flexion in the stance limb were found during dual-task obstacle-crossing (Figs 2–5 and Tables 2 and 4).

No interactions were observed between the task and obstacle height factors for any of the variables. With increasing obstacle height, healthy older adults during single- and dual-task showed linearly reduced their crossing speeds and leading heel-obstacle distance, but linearly increased their leading and trailing toe-obstacle clearances (Table 1). When the leading toe crossed above the obstacle, whether during single task or dual task, the subjects linearly increased the pelvic anterior tilt, upward list and contralateral rotation, hip flexion and abduction, knee flexion, ankle dorsiflexion of the swing limb and ankle abduction of the stance limb, but linearly decreased the hip adduction, ankle dorsiflexion of the stance limb (Tables 2 and 3). On the other hand, when the trailing toe crossed above the obstacle, healthy older adults during single and dual task linearly increased the pelvic posterior tilt, contralateral rotation, hip flexion, and ankle dorsiflexion of the swing limb, but decreased the pelvic upward list and hip adduction, knee flexion and ankle abduction of the stance limb (Tables 2 and 4).

## Discussion

The current study aimed to quantify the pelvis-leg kinematic adjustments of the lower limbs during cognitive-motor obstacle crossing in healthy older adults by comparing single-task and dual-task conditions. During dual-task crossing, the healthy older adults showed decreased crossing speeds and specific kinematic adjustments at the pelvis, hip and knee joints to increase both leading and trailing toe-obstacle clearances compared to single-task condition. The current results suggest that divided attention prompted the older adults to alter the pelvis-leg kinematics for a more conservative crossing strategy to reduce tripping risks. However, previous studies have shown that increased toe-obstacle clearance may increase the challenge of maintaining whole body balance, exposing to a greater risk of loss of balance [47]. Considering the overall risk of falls, it is suggested that older adults should consider balance training and avoid distractions, such as phone use during obstacle-crossing, to mitigate fall risks.

The observed increase in the toe-obstacle clearances during dual-task obstacle-crossing was closely associated with kinematic adjustments of the pelvis and the lower limbs. At leading-limb crossing, dual task led to increased abduction at the swing hip, increased flexion at the swing knee and decreased adduction at the stance hip, all contributing to the noted rise in the toe-obstacle clearance (Fig 3 and Tables 2 and 3). The increased anterior tilt of the pelvis tended to decrease the toe-obstacle clearance but was not big enough to diminish the increase induced by the other kinematic adjustments. At trailing-limb crossing, an increase in trailing toe-obstacle clearance was attributed to the increased swing knee flexion and decreased pelvis upward list, knee flexion and hip adduction in the stance limb despite the opposite tendency of the increased pelvic posterior tilt (Fig 4 and Tables 2 and 4).

Obstacle crossing is more neuromechanically challenging than walking, with greater ranges of motion of the lower limb joints and challenges in endpoint control, while both tasks require controls from the higher functions of the CNS. Compared to walking, additional angular changes at the joints may be necessary for the stance limb to ensure stability, while the swing limb achieves the required mobility precision control of the end-point when crossing the obstacle. While the older adults were able to cross obstacles with increased toe-obstacle clearances and thus reduced risk of tripping under divided attention, this was achieved at a cost. Increased leading and trailing toe-obstacle clearances are accompanied by necessary posture adjustments, which may place greater challenges on body balance, compromise dynamic balance control, and lead to greater instability [47–49], resulting in greater excursion of the body’s COM and the COM-COP separation [47,48].

The observed kinematic changes are likely related to the dual-task interference between cognition and motor performance when performing cognitive-motor dual task [50,51]. A previous study shows that cognitive-motor dual-task walking is linked to altered activation in the indirect locomotor pathway and the frontoparietal network [52], which are involved in attention, working memory and executive functions. Executive functions encompass cognitive processes such as planning, prioritisation, cognitive inhibition, decision-making, or cognitive control of behaviour [53]. The current study has found that performing an obstacle crossing task while dual-tasking affects the brain in ways that change how high the toes are lifted [6,22] and the overall gait kinematics. These changes are linked to the high-level cognitive areas of the brain. Increased toe-clearance during challenging obstacle-crossing tasks could result from the difficulty of dividing attention between the physical task and a concurrent cognitive task, leading to less efficient performance (known as a cognitive-motor dual task). This issue might be more pronounced in older adults with cognitive challenges, like mild cognitive impairment (MCI). Further research focusing on MCI patients is warranted to gain a deeper understanding of these dynamics.

According to the capacity limit theory, as more resource is allocated to one task, less resource is available for the other task [54]. During a cognitive-motor dual task, due to the limited amount of resources that can be allocated to simultaneous tasks, the cognitive demands relative to the individual’s cognitive capacity influence the combined performance. If the cognitive load of performing two tasks simultaneously exceeds an individual’s cognitive capacity, then performance on one or both tasks may deteriorate. The current study demonstrated that serial subtraction, as a cognitive task, elicited kinematic adaptations during obstacle crossing in older adults. It is expected that more challenging tasks, such as those requiring verbal fluency, executive control, or phone use (e.g., texting and reading), may have an even greater negative impact on motor performance and increase the risk of falls [55,56]. Further study on obstacle crossing while using mobile phones will be necessary to investigate the extent to which gait adaptations vary across different dual-task types. The current study highlights that cognitive-motor dual task may prompt healthy older adults to adjust their pelvis-leg kinematics for a more conservative crossing strategy, with increased toe clearance, thereby reducing tripping risks. However, excessive toe-obstacle clearance can also increase the challenge of maintaining whole-body balance, thereby elevating the risk of losing balance [57]. Understanding how dual task alters balance-control strategies is valuable for refining rehabilitation programs that incorporate obstacle crossing under dual-task paradigms. In addition, the current gait metrics could be particularly valuable when used in machine learning models to classify altered gait patterns and detect increased risk in real time using wearable sensors, thereby guiding the design of gait-monitoring and fall-alert systems.

The current study was the first to quantify pelvis-leg kinematic adjustments in healthy older adults during cognitive-motor obstacle crossing by comparing single-task and dual-task conditions. The protocol used anticipated obstacles; therefore, the observed strategy of increased toe-obstacle clearance may not generalise to unanticipated obstacles. Future work should test whether similar adjustments occur when obstacles are unexpected under dual-task conditions. Characterising age differences was not an objective of the study; hence, a young-adult cohort was not included. Nonetheless, the current approach can be used to quantify age-related differences in compensatory mechanisms by prospectively comparing young and older cohorts, and to identify the effects of divided attention on kinematic adjustments during obstacle crossing in individuals with mild cognitive impairment or in other populations at increased fall risk. The effects of motor and cognitive dual task on whole-body balance control also merit further investigation.

## Conclusions

The current study has demonstrated that dual-tasking during obstacle crossing significantly alters the kinematics of the pelvis-leg apparatus in healthy older adults. While undertaking dual tasks, participants exhibited a reduction in crossing speed and an increase in toe-obstacle clearances, suggesting an adaptation to prioritize safety over speed, thereby reducing the likelihood of tripping. These behavioural adaptations were accompanied by distinct kinematic changes at the pelvis, hip, and knee joints, which varied depending on whether the leading or trailing toe was crossing the obstacle. Despite the potential benefit of reduced tripping risk, the increased toe clearances may inadvertently elevate the challenge to whole-body balance, signalling a complex trade-off between obstacle negotiation and balance maintenance. Considering the overall risk of falls, it is suggested that older adults should consider balance training and avoid distractions, such as phone use during obstacle-crossing, to mitigate fall risks. Future studies may extend this research to scenarios involving unexpected obstacles and further evaluate the overall impact of cognitive-motor dual task on postural control in patients with MCI or populations with increased fall risks.
